# Relationships between Athletic Motor Skill Competencies and Maturity, Sex, Physical Performance, and Psychological Constructs in Boys and Girls

**DOI:** 10.3390/children9030375

**Published:** 2022-03-08

**Authors:** Ben J. Pullen, Jon L. Oliver, Rhodri S. Lloyd, Camilla J. Knight

**Affiliations:** 1 Youth Physical Development Centre, Cardiff Metropolitan University Cyncoed Campus, Cardiff CF23 6XB, UK; b.pullen@outlook.cardiffmet.ac.uk (B.J.P.); joliver@cardiffmet.ac.uk (J.L.O.); 2 Welsh Institute of Performance Science, Sport Wales, Sophia Gardens, Cardiff CF11 9SW, UK; c.j.knight@swansea.ac.uk; 3 Sports Performance Research Institute, New Zealand (SPRINZ), AUT University, Auckland 0632, New Zealand; 4 Centre for Sport Science and Human Performance, Waikato Institute of Technology, Hamilton 3200, New Zealand; 5 School of Sport and Exercise Sciences, Swansea University, Fabian Way, Swansea SA1 8EN, UK

**Keywords:** physical literacy, strength and conditioning, youth

## Abstract

The purpose of this study was to examine the relationships between athletic motor skill competencies (AMSC), maturation, sex, body mass index, physical performance, and psychological constructs (motivation to exercise, physical self-efficacy, and global self-esteem). Two-hundred and twenty-four children aged 11–13 years old were included in the study and sub-divided by sex. The athlete introductory movement screen (AIMS) and tuck jump assessment (TJA) were used to assess AMSC, while standing long jump distance assessed physical performance. Online surveys examined participants’ motivation to exercise, physical self-efficacy, and self-esteem. Trivial to moderate strength relationships were evident between AMSC and BMI (boys: *r_s_* = −0.183; girls: *r_s_* = −0.176), physical performance (boys: *r_s_* = 0.425; girls: *r_s_* = 0.397), and psychological constructs (boys: *r_s_* = 0.130–0.336; girls *r_s_* = 0.030–0.260), with the strength of relationships different between the sexes. Higher levels of AMSC were related to significantly higher levels of physical performance (*d* = 0.25), motivation to exercise (*d* = 0.17), and physical self-efficacy (*d* = 0.15–0.19) in both boys and girls. Enhancing AMSC may have mediating effects on levels of physical performance and psychological constructs in school-aged children, which may hold important implications for physical activity levels and the development of physical literacy.

## 1. Introduction

The development of muscular strength and movement skills in youth is a key outcome for leading health authorities, long-term athletic development models, and physical education curricula [1,2,3]. Reduction of chronic diseases, reduced injury risk, improved health and well-being, and longevity in physical activity are all products of improving muscular strength and movement skills in youth [4,5,6,7,8,9]. Previous research investigating the development of movement skills has focused mainly on fundamental movement skills in youth, which include locomotive, stabilisation, and manipulation skills [6,7,10,11,12,13,14]. Lloyd et al. [15] introduced the concept of athletic motor skill competencies (AMSC) to guide strength and conditioning coaches and physical education teachers in developing young people’s athleticism. The AMSC are considered foundational movement skills that underpin athletic movements, providing youth with the skills needed to safely and effectively participate in physical activity with confidence and competence [15,16]. The AMSC incorporate skills such as lower-body bilateral and unilateral movements, upper-body pushing and pulling, anti-rotation and core bracing, acceleration, deceleration and reacceleration, jumping, landing and rebounding mechanics, and traditional fundamental movements skills [15,17,18]. To assess the AMSC, movement screening can be implemented, using process orientated measures to assess movement limitations and biomechanical deficits in individual movements against pre-determined performance criteria [19]. 

The AMSC are vital to the habitual development of athleticism in youth, and central to long-term athletic development [16]. Importantly, a recent editorial has highlighted the need for the AMSC to be considered a central aim in physical education [20]. In the UK, the four key aims of physical education are to develop competence to excel in a broad range of physical activities, get children active for sustained periods of time, engage in competitive sports, and lead healthy, active lives [21]. Despite these aims, children in the UK currently do not meet recommended daily physical activity guidelines [22], and participation in a game/sports curriculum does not ensure the development of muscular strength, physical fitness, or AMSC [15]. Globally, only a small proportion (20%) of adolescents are hitting daily recommended physical activity guidelines [22]. The World Health Organization has identified national success stories in the quest of promoting physical activity through the education sector or in schools [23]. For example, in Denmark, 45 min of daily physical activity was introduced in 2013. Finland introduced the “Finnish schools on the move” initiative, which was piloted in 2010–2012 with schools implementing their own plans to increase physical activity during lessons, school breaks, and after school. The UK was recognised for the daily mile initiative, which was launched in 2017 and aimed to encourage children to run or jog for at least 15 min every day.

One key determining factor for health outcomes and levels of physical activity in children is the concept of physical literacy, which is underpinned by motor competence, motivation, and confidence [24,25]. Previous research in physical literacy has identified that two current paradigms exist [25]. The first paradigm, based on research by Whitehead, considers physical literacy as a holistic ability, with individuals being able to skilfully interact with their ever changing environments with motivation, confidence, and physical competence [26,27]. The second paradigm, the long-term athletic development approach, focuses solely on the physical components of physical literacy [28]. However, the paradigms should not be considered mutually exclusive. In this study we consider physical literacy to be fluid and flexible with no definitive boarders, somewhat ambiguous, akin to the pluralist view of concepts as in the recent Young et al. [29] paper. Thus, there is a need to consider relationships between AMSC and psychological constructs.

Little is known about the associations between AMSC and psychological constructs in school children and if interactions differ between the sexes. In a population of Australian adolescent school children (14 ± 0.5 years), movement screen scores demonstrated significant correlations to resistance training self-efficacy and motivation to exercise [30]. In younger children (6–7 years), 10 weeks of strength and conditioning focusing on the development of movement resulted in significant improvements in physical self-efficacy in boys but not girls [31]. However, it is still unknown whether children possessing higher levels of AMSC and psychological constructs differ from those scoring lower in AMSC movement screens and if variations between the sexes exist. To understand the importance of the AMSC in the holistic development of youth, the interaction between affective themes such as confidence, motivation, and perceptions of physical competence merit investigation [25]. Intrinsic motivation is related to affective change during both moderate and hard intensity exercise in adolescents and plays an important role in motor performance and learning [32,33]. Physical self-efficacy (an individual’s situational beliefs in their own abilities [34]) is a significant precursor to physical activity with mediating effects on motivation [35]. Investigations into school children need to consider boys and girls separately, as factors such as maturation timing, physical growth, and psychological constructs vary between the sexes [36,37,38]. Differences in the rate and timing of growth between the sexes become most prominent with the onset of the pubertal growth spurt, with large divergences between boys and girls impacting physical performance [39,40].

Previous research has identified associations between AMSC and muscular strength in school children, however such studies have been exclusive to same sex comparisons [41,42]. To the authors’ knowledge, only one study has previously investigated the relationship between AMSC, muscular fitness, and psychological constructs [30]. The authors focused on the prevalence and correlates of AMSC in school children, revealing significant associations between muscular fitness and AMSC (males: β = 0.31, *p* ≤ 0.001; females β = 0.33, *p* ≤ 0.001), but AMSC associations to autonomous motivation were only small in magnitude (β ≤ 0.2). However, it is still unknown whether higher levels of AMSC demonstrate associations to BMI, physical performance, and psychological constructs. It is hypothesised that the AMSC may have relationships with physical performance and psychological constructs with variations between the sexes. Therefore, the aims of this study were to (1) examine the relationships between maturity and AMSC, BMI, physical performance and psychological constructs, and (2) identify differences between boys and girls and higher and lower levels of AMSC.

## 2. Materials and Methods

### 2.1. Experimental Overview

To examine the relationship between AMSC and psychological constructs and lower body strength in school children, a cross-sectional design was implemented. The testing included movement screens assessing AMSC via the Athlete Introductory Movement Screen (AIMS), physical performance via a standing long jump assessment to investigate lower limb strength and surveys to examine motivation to exercise, physical self-efficacy, and global self-esteem. Participants were recruited from two schools in areas of lower socio-economic status in South Wales [43]. The school principal or head of physical education granted pupil consent via in-loco-parentis. Prior to the start of the study, children’s parents were provided with information letters and then given the opportunity to opt their child out of the study, and participant assent was obtained from all pupils. Testing for the study was performed in a physical education lesson at each school, supervised by the primary researcher and assisted by three other paediatric exercise science PhD students. Testing sessions were performed during the school term of the year, between September and October 2018. Prior to the study, ethical approval was granted by Cardiff Metropolitan University ethics committee (17-10-01R). 

### 2.2. Participants

Boys and girls in school years 7 and 8 (11–13 years old) from two schools were invited to participate in the study. The first two school years of secondary education were selected to target children before going through the secondary school system, at an age sensitive to physical and psychological changes when physical activity levels decline [7,39]. A total of 257 participants were initially recruited and after some participants opted out, 224 school children were included in the study (boys = 119; girls = 105). Table 1 provides the age, estimated maturity, anthropometrics, and body mass index (BMI) for the male and female participants included in the study. Estimated maturity was calculated by using sex-specific regression equations according to the methods proposed by Mirwald et al. [44], which have been shown to possess an error of approximately 6 months.

### 2.3. Athletic Motor Skill Competencies

At the start of the testing session, all participants performed a standardised five minute warm up. To assess multiple AMSC, The Athlete Introductory Movement Screen (AIMS) [45] was used to examine pupils’ competency in lower body bilateral and unilateral, core bracing and anti-rotation, and upper body pushing, as suggested by Pullen et al. [19]. Although there are numerous movement screens, the AIMS is regarded the best at assessing AMSC in youth populations [19], therefore AIMS scores represent the concept of AMSC in this study. The Tuck Jump Assessment (TJA) [46] was also implemented to assess jumping, landing, and rebounding competency. Both screens have demonstrated intra-rater intraclass correlation coefficients >0.8, indicating acceptable reliability in youth populations [45,47]. 

Standardised demonstrations of the movements in the AIMS (overhead squat, lunge, brace with chest touch, and push-up) were provided and details regarding the start, midpoint, and end positions of movements were given to participants. No questions were answered regarding technique, nor was cueing provided to pupils during the screening. Participants were given the opportunity and encouraged to perform a practice set of each movement (four repetitions) as performed in the screen. All movements in the AIMS were performed for two sets of four repetitions, except for the unilateral movements (lunge and the front support brace with chest touch) whereby participants performed two repetitions each side. The AIMS implements a segmental analysis, scoring four segmental criteria for each movement over multiple repetitions [19]. Each of the four criteria is awarded 1–3 points: 3 points = full proficiency and consistency, 2 = moderate proficiency and/or inconsistent performance, 1 = poor/and/or inconsistent movement ability [45]. For each movement, there is a maximum of 12 points and a minimum of 4 points available. Thus, total scores between 16–48 are achievable for the AIMS; higher scores are indicative of higher levels of AMSC. All performance criteria of the AIMS are provided in Table 2.

Participants performing the AIMS were filmed individually during the testing session to be retrospectively graded by the primary researcher; allowing for movements to be paused, slowed down and replayed to improve grading accuracy [19]. Cameras (iPad Mini 2, Apple, Cupertino, CA, USA) were placed five meters from the participants at a height of 0.7 m. Participants performed the first four repetitions in the frontal view, then rotated 90 degrees to face the sagittal view to perform the second set of four repetitions.

Prior to performing the TJA, participants were provided a standardised demonstration and instructed to perform repeated jumps in place and as high as possible continuously for a total of 10 s. Participants were instructed to spend as little time as possible between jumps and to pull their knees high up to their chest during each jump. The TJA is graded out of 10, with one point awarded for each movement deficit detected, thus a higher score reflects poorer movement quality. The performance criteria used in this study are provided in Table 3. 

### 2.4. Intra-Rater Reliability of AMSC Screens

The intra-rater reliability of the primary researcher was determined for both the AIMS and TJA. The recorded trials of 20 participants performing the AIMS were selected at random to be included for analysis. Videos were graded on three separate occasions by the primary researcher, with two weeks in-between each grading to remove the chance of recalling previous participant scores. All scores were kept separate by the primary researcher until analyses were run to determine reliability. The same protocol was previously used by the primary researcher for ten participants completing the TJA, demonstrating excellent intra-rater reliability in a sample of secondary school children (ICC = 0.91) [48].

### 2.5. Physical Performance

To assess lower limb power, participants performed the standing long jump assessment, an assessment commonly used in youth populations to assess musculoskeletal fitness [49]. Participants were provided with a standardized demonstration and instructions for performing the long jump. Participants’ feet had to be behind the baseline prior to the jump and remain in the landing position for a jump to be counted. The tape measure was placed parallel to the jumping area, and jump length was determined by the distance between the baseline and the heel from where participants landed in centimeters. The standing long jump assessment has previously demonstrated excellent reliability in youth populations (ICC = 0.94) and is linked to health-related musculoskeletal fitness [50,51].

### 2.6. Psychological Measures

Whilst maintaining questionnaire structures and scales, all questionnaires were converted into an online software (quicktapsurvey.com) allowing for interactive survey collection on mobile tablets (iPad Mini 2, Apple Cupertino, CA, USA) from participants. Survey results were then downloaded by the primary researcher once the testing session had finished. All participants were instructed to answer what they felt was their first choice naturally and not to consider what they believe maybe the right answer if an adult was scoring the questionnaires. The primary researcher would answer any questions regarding the scale and structure of the questionnaire but nothing pertaining to which answer to choose. 

### 2.7. Motivation to Exercise

To assess participant motivation to exercise, the Behavioural Regulation in Exercise-2 [52] was used. The questionnaire assesses the five subscales of motivation (intrinsic, identified, introjected, external, amotivation) aligned with the self-determination theory [53]. From the questionnaire, a Relative Autonomy Index (RAI) is formed, with a higher RAI indicative of higher levels of autonomous motivation. Items are structured similarly to the following examples “I enjoy my exercise sessions” or “I think exercising is a waste of time”. Items are scored on a five-point Likert scale (0–4) from “Not true for me (0)” to “Very true for me (4)”. Reliability of the questionnaire has been demonstrated across all five of the subscales of motivation in an adolescent population (Cronbach alpha > 0.8) [54]. 

### 2.8. Physical Self-Efficacy

The Perceived Physical Ability Scale for Children [55] was used to assess physical self-efficacy. This scale is comprised of six items, with responses detailed on a Likert scale from 1–4, such as 4 = “I move very rapidly”, 3 = “I move rapidly” 2 = “I move slowly” 1 = “I move very slowly”. In total, a score of 24 is achievable, with higher scores indicative of higher levels of physical self-efficacy [55]. The questionnaire has demonstrated good levels of reliability in young children (Cronbach alpha = 0.72).

### 2.9. Global Self-Esteem

Global self-esteem was measured using the Rosenberg Self-Esteem Scale [56]. This scale was chosen due to its demonstrated reliability (Cronbach alpha = 0.85) [57] and previous implementation in adolescent research [56,58]. Responses are provided on a 4-point Likert scale (0–3) from “strongly agree” (3) “to “strongly disagree” (0), with questions framed as follows “I take a positive attitude towards myself”. For questions 2, 5, 6, 8 and 9 the scoring is reversed, whereby “strongly agree” = 0, and “strong disagree” = 3. 

### 2.10. Statistical Analysis

All data were analyzed using Microsoft Excel Version 16.30 and IBM SPSS statistics version 24 (IBM corp, Armonk, NY, USA). To establish the intra-rater reliability for both the AIMS and TJA movements in the screen and the total screen score, the intra-class correlation coefficient (ICC) was calculated. To further investigate the reliability of each individual performance criteria for each segment in each screen, percentage agreement and the Kappa statistic (*k*) were calculated. The ICC and Kappa strength of agreement were interpreted as the following: 0, no agreement; 0.01–0.20, slight agreement; 0.21–0.40, fair agreement; 0.41–0.60, moderate agreement; 0.61–0.80, substantial agreement; 0.81–1.00, almost perfect agreement [59]. All data were categorized and analyzed by sex. 

To determine the normality of data distribution, Shapiro–Wilk tests were implemented on all measures. For normal and non-normal distribution data, parametric and non-parametric analyses were implemented, respectively. For normally distributed data, averages are presented as mean ± standard deviation and for non-normal data, averages are presented as medians and interquartile ranges. To investigate the relationship between variables, a Spearman’s correlation (*r_s_*) was used to identify statistically significant correlations within each sex. The strengths of correlation coefficients were interpreted as: trivial (0.0–0.1), small (0.11–0.3), moderate (0.31–0.5), large (0.51–0.7), very large (0.71–0.9), nearly perfect (0.91–0.99), and perfect (1.0) according to previously published guidelines [60]. 

Mann–Whitney *U* tests were implemented to determine the difference between boys and girls in AMSC measures, physical performance, and psychological constructs. To compare the AMSC associations to psychological constructs, participants were split by sex and then into lower and higher competency groups using the median split based on AIMS total scores. Participants who fell on the median were removed from the analysis. Mann–Whitney *U* tests were applied to identify the difference between higher versus lower AMSC groups on physical performance and psychological constructs. Effect sizes were calculated to establish the magnitude of the difference between the sexes and competency groups according to Cohen’s *d* statistics, exercising the following thresholds: <0.2 (trivial), 0.2–0.59 (small), 0.6–1.19 (moderate), 1.20–1.69 (large) and >1.70 (very large). The differences between higher and lower AMSC groups in both males and females were reported as percentage difference, effect size (*d*), and statistical significance. Standard alpha level was set at *p* < 0.05 for all analyses.

## 3. Results

### 3.1. Intra Rater Reliability

Intraclass correlation coefficients demonstrated good reliability for the total AIMS score (ICC = 0.86) and moderate to good reliability for individual movements in the screen (ICC: overhead squat = 0.89; lunge = 0.82; front support brace + shoulder tap = 0.64; push-up = 0.76). The typical error for total AIMS score was 1.57 and the typical error for individual movements ranged from 0.58–0.85. The mean Kappa (k) statistic for the four criteria of each movement ranged from slight to substantial agreement (0.054 to 0.687) with the percentage agreement ranging from 69% to 84% for segmental analysis reliability in the AIMS. Most criteria (14/16) demonstrated moderate to perfect reliability (k = 0.41–1.00), with two demonstrating fair or no reliability. The TJA demonstrated excellent reliability (ICC = 0.91) with individual performance criteria kappa statistic indicating poor to almost perfect agreement (k = 0.286 to 1.000) with the percentage agreement ranging from 50% to 100%. 

### 3.2. Relationships

Characteristics of individual movements and totalled movement screen scores, lower limb strength and motivation to exercise, physical self-efficacy and global self-esteem of both males and females are displayed in Table 4. The only difference in AMSC was exhibited in the push-up movement, with males scoring significantly higher than females (*d* = 0.28). Females also demonstrated significantly lower physical self-efficacy (*d* = 0.21) and global self-esteem levels than males (*d* = 0.20).

Maturity offset was moderately correlated to BMI in both males and females. In boys, BMI had small associations to AIMS and tuck jump assessment and moderate associations to standing long jump. Whilst in the females, a small association between BMI and standing long jump was present with non-significant relationships to AIMS and tuck jump assessment. In boys, BMI demonstrated small correlations to motivation to exercise and global self-esteem, whereas in girls, BMI displayed small relationships to motivation to exercise and global self-esteem and a moderate relationship to physical self-efficacy. All Spearman’s Rho correlations between BMI maturity offset, AMSC movement screen scores, and psychological constructs are displayed in Table 5.

### 3.3. Difference between Boys and Girls

**Figure 1:**
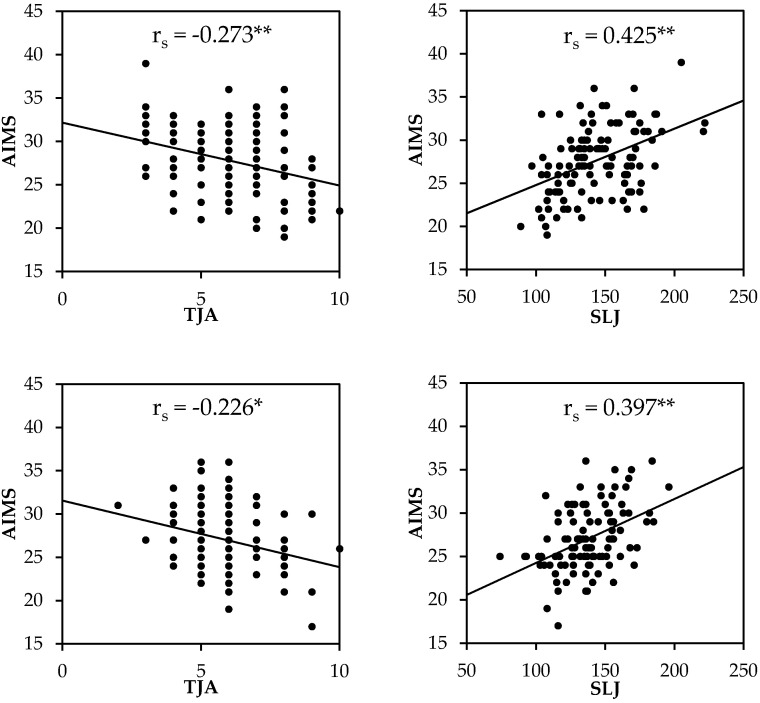
Associations between athletic motor skill competencies via the Athlete Introductory Movement Screen (AIMS), tuck jump assessment (TJA), and standing long jump (SLJ) performance in boys (above) and girls (below). ** Correlation is significant at the *p* < 0.01 level. * Correlation significant at the *p* < 0.05 level.

**Figure 2:**
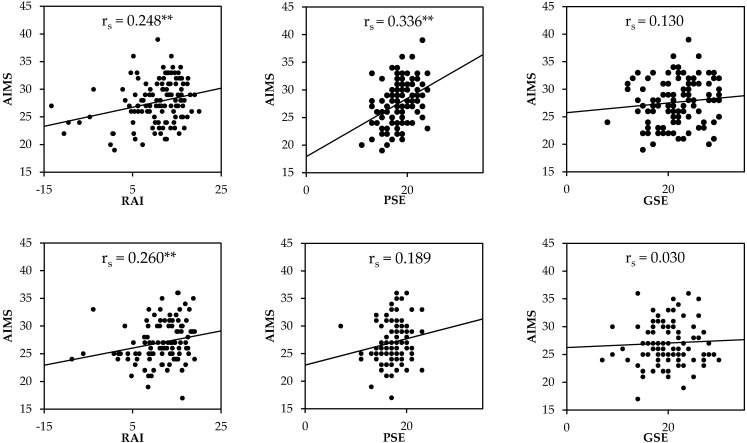
Associations between athletic motor skill competencies via the Athlete Introductory Movement Screen (AIMS), motivation to exercise (RAI), physical self-efficacy (PSE), and global self-esteem (GSE) in males (above) and females (below). ** Correlation is significant at the *p* < 0.01 level.

The AIMS demonstrated a small–moderate strength of relationship between TJA and SLJ, motivation to exercise, and physical self-efficacy (*r_s_* = 0.212 to −0.308) in males whereas the female participants demonstrated no significant correlations between any measure (*r_s_* = >0.154) and AIMS except for the TJA (*r_s_* = −0.226). In both sexes, the strongest association with AIMS was standing long jump performance (males: *r_s_* = 0.425; females: *r_s_* = 0.397). In males, there were moderate correlations between AIMS and TJA, standing long jump, and physical self-efficacy. Conversely, in females only standing long jump distance was moderately correlated with AIMS. BMI was moderately correlated to standing long jump performance in males and physical self-efficacy and global self-esteem in females. The relationships between AIMS, maturity, BMI, standing long jump performance, TJA, and psychological measures are displayed in Figure 1 and Figure 2 for both males and females.

### 3.4. High and Low AMSC Comparison

There were significant differences between higher and lower AMSC groups in tuck jump assessment, standing long jump, motivation to exercise and physical self-efficacy in both boys and girls. There were differences in BMI between high and low competency groups in both boys and girls, however the difference only neared significance (*p* = <0.06). Between-group comparisons for each of the independent variables for both higher and lower competency groups are displayed for boys and girls in Table 6 and Table 7, respectively.

## 4. Discussion

This study examined the relationship between AMSC movement screen scores, sex, maturity offset, BMI, physical performance, and selected psychological constructs in boys and girls. The findings indicate that the ability to perform AMSC in the AIMS are positively and significantly correlated to physical performance, motivation to exercise, and physical self-efficacy in both male and female children. However, it should also be recognized that where significant relationships were observed, they were small to moderate in strength. When grouped according to the composite AIMS score, higher competency boys and girls jumped significantly further, were more motivated to exercise, and possessed greater physical self-efficacy than those with lower AIMS scores. Cumulatively, the findings indicate some important association between AMSC movement screen scores, physical performance, and psychological constructs in boys and girls. 

### 4.1. Relationships

Maturity offset demonstrated moderate associations with BMI in both males and females, which may be attributed to maturation as it is a time that sees major changes and variation between the sexes in growth and stature [37,39,61]. The only other relationship to maturity offset was a small correlation with standing long jump performance in females. Although only a small relationship in the present study, girls have been shown to experience a decrease in neuromuscular control over the course of maturation [62]. Previous research has demonstrated a maturation effect between movement competency assessments and jump performance measures [63,64]. Yet, such studies often sample participants with a large range in age and maturity, thereby increasing sample heterogeneity and potentially inflating the correlations [63,64]. Multiple internal factors can influence the development of AMSC, including growth, maturation, adolescent awkwardness, and neural development [62,65,66,67]. Nevertheless, the weak relationships between maturity status and AMSC may reflect the differential timings of the development of the central nervous system and movement competence [12,68,69]. It may then be that AMSC fail to naturally develop further in early adolescence unless the environment a child is exposed to provides an adequate stimulus. This reinforces the importance of well-designed physical education curricula to promote the continued development of AMSC as boys and girls move into secondary school. 

Associations between maturity status and BMI were present in both sexes, which may be attributed to the population being circa puberty, a period characterized by marked somatic changes [39,65,70]. There were small to moderate relationships between BMI, physical performance, and psychological constructs in boys and girls. Boys demonstrated stronger associations between BMI and AMSC-based assessments (AIMS and TJA), whereas BMI in girls was more strongly correlated to psychological constructs. The impact of BMI on adolescents’ self-perceptions is strongly influenced by their wider sociocultural context, which has previously been shown to influence girls more strongly than boys. [71] Furthermore, the female group was characterized by lower levels of physical self-efficacy and self-esteem in this study, which reflects previous findings that the greatest decline in body satisfaction in adolescents is experienced by females [72]. Longitudinal evidence has supported a negative association between weight status and movement competency [73,74]. In addition, an AMSC intervention conducted by Smith et al. [75] was successful in positively mediating body composition and muscular fitness. Despite the difference in BMI between higher and lower AMSC groups nearing significance (*p* < 0.06), the amount of shared variance between AMSC and BMI was relatively low (<20%), and thus associations should be interpreted with caution. 

The standing long jump assessment demonstrated the strongest associations with the AIMS in both boys (*r* = 0.425) and girls (*r* = 0.397). The standing long jump is an assessment of lower limb strength and requires individuals to rapidly produce force in the lower limbs while maintaining postural control. Many of the movements in the AIMS, namely the overhead squat, lunge, and the brace with shoulder touch require activation and control of the musculature surrounding the trunk, hip, knee, and ankle [76,77]. Therefore, the standing long jump and AIMS are likely underpinned by the same neuromuscular control mechanisms and physical qualities, which might explain the significant moderate correlations reported. Associations between the AIMS and TJA were not as strong in comparison to the standing long jump. Although the TJA uses similar muscle groups as movements identified in the AIMS, it requires repeated maximal efforts with higher magnitudes and rates of force development [78]. The repeated rapid maximal efforts with fatiguing elements to the TJA may explain the weaker associations to AIMS compared with the standing long jump.

### 4.2. Sex Differences

Boys and girls performed similarly across most AMSC measures, with competency in the push up the only difference between the sexes. This may be due to males out performing females in health-related fitness measures [79]. There were more consistent differences in psychological constructs, with boys having significantly greater physical self-efficacy and global self-esteem. The study population scored below the normative average in physical performance respective to age with males scoring in the 30th percentile and females in the 40th percentile for the SLJ [79]. Additionally, the population in this study scored below previous standards for AMSC (median AIMS score = 27–28) when compared with more athletic populations (mean AIMS score = 36) [45]. The findings in this study align with previous research which has demonstrated that AMSC are not well developed in children [30]. However, previous research has not considered the influence of AMSC on important psychological constructs linked to physical literacy. Additionally, previous research has not implemented movement screens as comprehensive as the AIMS at measuring movement consistency in the AMSC in youth [19]. To that end, previous research implementing certain screening protocols may need reconsideration.

### 4.3. Differences between High and Low AIMS Score

When boys and girls were categorized by AIMS score, relationships between higher and lower competency groups and psychological constructs were the same in both sexes. Higher AIMS scores equated to significantly higher physical performance, jumping landing and rebounding mechanics, motivation to exercise, and physical self-efficacy in both sexes. School children possessing higher AIMS scores were 5.3% and 5.6% more motivated to exercise, performed 11.5% and 12.2% better physically, and had 6.6% and 8.3% lower body mass in males and females, respectively. There were no significant associations between AIMS and global self-esteem in both males and females or the high- or low-competency groups. Global self-esteem shows substantial stability over time with increasing stability from childhood to adolescence in both sexes; consequently, changes in physical competence may not be enough to impact self-esteem alone at global level [80]. The current study builds on the absence of knowledge; establishing children’s physical performance differentiates AMSC ability with potential mediating effects on physical self-efficacy and motivation to exercise in school children.

### 4.4. Strengths

This study is the first of its kind to identify associations between AMSC and constructs from psychological domains in both boys and girls in a UK secondary school setting. The identified associations between the AMSC movement screen scores, motivation to exercise, and physical self-efficacy establish AMSC associations with affective themes within physical literacy [25]. Thus, the study’s findings present the potential importance of developing young people’s AMSC to aid in positive psychological development. This study also provides novel between-sex comparisons, providing strength and conditioning practitioners normative values in AMSC in school children which may benchmark future research and coaching. Furthermore, this study furthers the field’s knowledge regarding the difference in psychological constructs of children who possess higher or lower abilities to perform the AMSC.

### 4.5. Limitations

This study only sampled children from schools in areas of lower socio-economic status, a known factor influencing motor performance [81]. This study establishes relationships to AMSC, yet it is still unknown whether relationships between AMSC, sex, maturity, physical performance, and psychological constructs vary dependent on the socio-economic status of youth. Youth in areas of higher socioeconomic status may demonstrate higher levels of competency, as the population in this study scored below the 50th centile in physical performance [79]. In addition, this study sampled children aged 11–13 years old, however the development of AMSC could be established earlier as a potential intervention to counteract the drop in physical activity levels, which begin as young as 7 years old [82]. Thus, investigating AMSC in a more heterogeneous population with wide age ranges including childhood to late adolescence with a variety of socio-economic and cultural backgrounds could support knowledge surrounding the AMSC and their importance to youth. Intra-rater reliability of certain movements in the screen were moderate and some of the segmental analyses showed only slight agreement, which combined underlines the importance of determining an individual’s reliability at subjectively scoring movements. However, this study used protocols and measures that have shown to be valid and reliable in paediatric cohorts, and, in addition to the robust research design, this study contributes novel and rigorous findings to the paediatric literature.

### 4.6. Practical Recommendations

Body mass index has more of a mediating effect on AMSC then maturity status, therefore, interventions aiming to positively effect BMI may also enhance AMSC and other associated outcomes. Improving AMSC may have some associated benefits on increasing physical performance, physical self-efficacy, and motivation to exercise. However, it should be noted that as relationships were often only small to moderate it may also be necessary to develop each quality independently. Nevertheless, practitioners and teachers delivering physical activity interventions may consider strength training focusing on the development of AMSC to develop the psychological attributes of youth. Considering Young’s and Whitehead’s physical literacy connotations, the relationships between physical competence and affective psychological domains demonstrate the potential efficacy of enhancing AMSC to holistically develop physical literacy. 

## 5. Conclusions

This study aimed to investigate relationships between AMSC, sex, maturity, BMI, physical performance, and psychological constructs in school children. Whilst relationships to AMSC were only modest, higher levels of AMSC were significantly related to physical performance, physical self-efficacy, and motivation to exercise in both boys and girls. These findings may have future implications for physical activity and physical literacy research and applied practice regarding school children in areas of lower socioeconomic status. In turn, the development of AMSC may have future implications leading to further research and interventions aiding the central vision of leading health authorities [1,23,83]. 

## Figures and Tables

**Table 1:** Participant characteristics.

Characteristics	Male (*n* = 119)	Female (*n* = 105)
Age (years)	11.8 ± 1.6	11.8 ± 2.1
Height (cm)	152.8 ± 9.6	153.8 ± 7.4
Body mass (kg)	47.3 ± 13.0	48.9 ± 11.3
Body mass index (%)	20.1 ± 4.5	20.6 ± 4.0
Maturity offset (years from PHV)	−1.6 ± 0.8	0.2 ± 0.7

Notes: Characteristics expressed as means ± standard deviations; maturity offset = time in years pre-to-post peak height velocity.

**Table 2:** Segmental performance criteria adapted from the Athlete Introductory Movement Screen (AIMS) [45].

AMSC	Segment	3 Points	2 Points	1 Point
Overhead Squat	Heels	Heels remain on floor throughout 4 consecutive repetitions	3 appropriate repetitions	2 or less appropriate repetitions
	Depth	Thighs are at least parallel to the floor at the bottom of the movement throughout 4 consecutive repetitions	3 appropriate repetitions; OR near parallel for all repetitions	2 or less appropriate repetitions
	Bar (dowel) and trunk position	Maintains bar overhead with appropriate shoulder/thoracic extension and trunk angle without rotation, throughout 4 consecutive repetitions	3 appropriate repetitions; OR bar position appropriate but minor deviation from appropriate thoracic extension and trunk angle on all repetitions; OR poor bar position but with appropriate thoracic extension and trunk angle on all repetitions	2 or less appropriate repetitions
	Frontal plane alignment	Appropriate alignment, symmetry and control of hip/knee/ankle, including thighs, move symmetrically throughout 4 consecutive repetitions	3 appropriate repetitions; OR minor misalignment and/or asymmetry on all repetitions	2 or less appropriate repetitions
Push-Up	Upper body alignment/control	Head, back and hips are held in a straight line throughout the movement on 4 consecutive repetitions	3 appropriate repetitions; OR minor misalignment/slight loss of control of 1 segment (e.g., head) on all repetitions	2 or less appropriate repetitions
	Shoulder position and control	Shoulders are away from ears (not shrugged/moved closer to ears during movement) AND elbow positioning is directed slightly anterior, not flaring, in 4 consecutive repetitions	3 appropriate repetitions; OR minor misalignment/slight loss of control on all repetitions	2 or less appropriate repetitions
	Hand position	Hands are placed under the shoulders AND hands not repositioned in any repetitions	Minor mispositioning of hands relative to shoulders (less than 10 cm); OR 1 repositioning of hands	Poor initial positioning OR; 2+ repositioning of hands
	Depth	Body is lowered until elbows at 90 degree angle for 4 consecutive repetitions	3 appropriate repetitions to 90 degrees; OR near 90 degrees on all repetitions (±10 degrees)	2 or less appropriate repetitions
Lunge	Trunk control	Maintains neutral spine during full movement (out and back), no flexion/extension or rotation for 4 consecutive repetitions	3 appropriate repetitions; OR minor misalignment/slight loss of control on all repetitions	2 or less appropriate repetitions
	Depth	Knee of rear leg lowered with control until almost touching the floor (<10 cm) for 4 consecutive repetitions	3 appropriate repetitions; OR Near appropriate depth for all repetitions OR some weight acceptance on knee on 1 rep (i.e., visible touch down)	2 or less appropriate repetitions
	Frontal plane alignment	Appropriate alignment and control of knee/ankle throughout 4 consecutive repetitions	3 appropriate repetitions; OR minor misalignment on all repetitions	2 or less appropriate repetitions
	Hip/ pelvic control	Appropriate alignment and control of hips with neutral pelvis throughout movement on 4 consecutive repetitions	3 appropriate repetitions; OR minor misalignment (end range) on all repetitions	2 or less appropriate repetitions
Front Support Brace & Shoulder Touch	Foot contact	Both feet remain on the ground throughout 4 consecutive repetitions, no foot sliding	3 appropriate repetitions (1 foot lift only); OR no repetitions lifted, but feet slide on up to 2 repetitions	2 or less appropriate repetitions (feet lifted in 2 or more AND/OR feet slide in 3 or more)
	Full body alignment	Holds the total body plane in straight alignment through legs (knees fully extended), hips, shoulders and head, for 4 consecutive repetitions	3 appropriate repetitions; OR minor misalignment on all repetitions	2 or less appropriate repetitions
	Resists rotation	Minimal rotation of the pelvis/hip complex during all 8 repetitions while changing from 4 to 3 points of contact (approx. 10 cm is acceptable)	6 or more appropriate repetitions; OR minor rotation on all repetitions	6 or less appropriate repetitions
	Controlled arm movement	Chest touches performed in controlled manner with arms deliberately returned to floor following chest touch on all 8 repetitions	6 or more appropriate repetitions; OR minor loss of control on all repetitions	6 or less appropriate repetitions

Notes: AMSC = athletic motor skill competencies.

**Table 3:** Assessment criteria adapted from the Tuck Jump Assessment [46].

AMSC		Assessment Criteria
Tuck Jump	1	Lower extremity valgus at landing
	2	Thighs do not reach parallel (peak of jump)
	3	Thighs not equal side-to-side (during flight)
	4	Foot placement not shoulder-width apart
	5	Foot placement not parallel (front to back)
	6	Foot contact timing not equal
	7	Excessive landing contact noise
	8	Pause between jumps
	9	Technique declines prior to 10 s
	10	Does not land in the same footprint
		(excessive in-flight motion)

Notes: AMSC = athletic motor skill competencies.

**Table 4:** Characteristics of AMSC, strength, and psychological attributes.

Variable	Male (*n* = 119)		Female (*n* = 105)		Male versus Female		
	MDN	IQR	MDN	IQR	% Dif	*d*	*p*
**AMSC**							
Overhead Squat (/12)	6.0	(5.0–7.0)	6.0	(5.0–7.0)	0	−0.05	0.462
Lunge (/12)	6.0	(4.0–7.0)	6.0	(5.0–7.0)	0	−0.08	0.255
FSB and chest touch (/12)	8.0	(7.0–9.0)	8.0	(8.0–8.0)	0	−0.04	0.573
Push-up (/12)	8.0	(6.0–8.0)	6.0	(6.0–8.0)	−25.0	−0.28	<0.001 *
AIMS (/48)	28.0	(25.0–30.5)	27.0	(25.0–30.0)	−3.6	−0.09	0.174
Tuck jump (/10)	6.0	(5.0–7.0)	6.0	(5.0–6.0)	0	−0.14	0.039 *
**Physical performance**							
Standing long jump	140.0	(125.0–166.5)	136.0	(126.0–153.0)	−2.9	−0.1	0.121
**Psychological constructs**							
Motivation (RAI)	11.9	(7.6–14.7)	12.8	(8.8–15.3)	7.6	−0.11	0.11
Physical self-efficacy	18.0	(17.0–20.0)	18.0	(16.0–19.0)	0	−0.21	0.001 *
Global self-esteem	22.0	(19.0–25.0)	20.0	(17.0–23.0)	−9.1	−0.21	0.002 *

Note: AMSC = athletic motor skill competencies; FSB = front support brace; AIMS = athlete introductory movement screen; % dif = percentage difference; MDN = median; ES = effect size; RAI = relative autonomy index; IQR = interquartile range. Interquartile ranges expressed as the first and third quartile. * Correlation significant at the 0.05 level.

**Table 5:** Spearman’s Rho correlations between body mass index, maturity offset, athletic motor skill competencies, and psychological constructs.

Variable	Maturity Offset		BMI	
	Male	Female	Male	Female
BMI	0.367 **	0.398 **		
AIMS	−0.066	0.035	−0.183 *	−0.176
TJA	0.024	0.098	0.282 **	0.060
SLJ	0.107	0.216 *	−0.359 **	−0.209 *
RAI	−0.043	0.009	−0.125 *	−0.283 **
PSE	0.009	−0.090	−0.157	−0.411 **
GSE	−0.056	−0.047	−0.200 *	−0.283 **

Notes: BMI = body mass index; AIMS = athlete introductory movement screen; TJA = tuck jump assessment; SLJ = standing long jump; RAI = relative autonomy index; PSE = physical self-efficacy; GSE = global self-esteem. ** Correlation is significant at the 0.01 level. * Correlation significant at the 0.05 level.

**Table 6:** Differences in characteristics between groups based on AIMS score in boys.

	Male						
Variable	Higher (*n* = 50)	IQR	Lower (*n* = 59)	IQR	% Difference	*d*	*p*
AIMS	31.00	(29.25–33.00)	25.00	(23.00–26.50)	−19.4	−0.60	<0.001 *
Maturation	−1.78	(−2.15–1.30)	−1.48	(−2.34–0.94)	17.0	0.07	0.284
BMI	18.38	(16.18–20.93)	19.60	(16.77–23.64)	6.6	0.13	0.053
TJA	6.00	(5.00–7.00)	7.00	(6.00–8.00)	16.7	0.16	0.018 *
SLJ	148.00	(135.50–171.00)	131.00	(115.50–159.00)	−11.5	−0.25	<0.001 *
RAI	12.96	(10.54–14.81)	10.75	(5.88–13.96)	−17	−0.17	0.012 *
PSE	19.00	(18.00–21.00)	18.00	(16.00–19.00)	−5.3	−0.19	0.005 *
GSE	23.00	(20.00–25.00)	21.00	(18.00–23.50)	−8.7	−0.12	0.071

Notes: AMSC = athletic motor skill competencies; Maturation offset = time in years pre- to post-peak height velocity; BMI = body mass index; TJA = tuck jump assessment; SLJ = standing long jump; RAI = relative autonomy index; PSE = physical self-efficacy; GSE = global self-esteem; IQR = interquartile range; *d* = effect size. * Correlation significant at the 0.05 level.

**Table 7:** Differences in characteristics between groups based on AIMS score in girls.

	Female						
Variable	Higher (*n* = 39)	IQR	Lower (*n* = 52)	IQR	% Difference	*d*	*p*
AIMS	31.00	(29.00–32.50)	25.00	(23.00–25.00)	−19.4	−0.59	<0.001 *
Maturation	0.29	(0.07–0.68)	0.33	(−0.14–0.79)	13.4	0.04	0.955
BMI	19.15	(17.58–20.76)	20.74	(18.36–23.56)	8.3	0.13	0.055
TJA	5.00	(5.00–6.00)	6.00	(5.00–7.00)	20	0.15	0.024 *
SLJ	152.00	(134.00–162.50)	133.50	(116.00–141.25)	−12.2	−0.25	0.001 *
RAI	14.83	(11.21–16.58)	11.63	(7.44–14.46)	−21.6	−0.17	0.008 *
PSE	18.00	(17.00–19.50)	17.00	(15.00–18.25)	−5.6	−0.15	0.029 *
GSE	21.00	(18.00–22.00)	20.00	(17.00–23.00)	−4.8	−0.03	0.607

Notes: AMSC = athletic motor skill competencies; Maturation = time in years pre- to post-peak height velocity; BMI = body mass index; TJA = tuck jump assessment; SLJ = standing long jump; RAI = relative autonomy index; PSE = physical self-efficacy; GSE = global self-esteem; IQR = interquartile range; *d* = effect size. * Correlation significant at the 0.05 level.

## Data Availability

Written informed consent has been obtained from subjects to publish this paper.
